# Aminoclay Nanoparticles Induce Anti-Inflammatory Dendritic Cells to Attenuate LPS-Elicited Pro-Inflammatory Immune Responses

**DOI:** 10.3390/molecules27248743

**Published:** 2022-12-09

**Authors:** Hyun Jung Park, Sung Won Lee, Jae Geun Song, Luc Van Kaer, Jae Hee Cheon, Soo-Jeong Lim, Hyo-Kyung Han, Seokmann Hong

**Affiliations:** 1 Department of Integrative Bioscience and Biotechnology, Institute of Anticancer Medicine Development, Sejong University, Neungdong-ro 209, Gwangjin-gu, Seoul 05006, Republic of Korea; 2 College of Pharmacy, Dongguk University-Seoul, Dongguk-ro 32, Ilsan-donggu, Goyang 10326, Republic of Korea; 3 Department of Pathology, Microbiology and Immunology, Vanderbilt University School of Medicine, Nashville, TN 37232, USA; 4 Department of Internal Medicine, Institute of Gastroenterology, Yonsei University College of Medicine, Yonsei-ro 50-1, Seodaemun-gu, Seoul 03722, Republic of Korea; 5 Department of Integrative Bioscience and Biotechnology, Sejong University, Neungdong-ro 209, Gwangjin-gu, Seoul 05006, Republic of Korea

**Keywords:** aminoclay, dendritic cells, lipopolysaccharide (LPS), IL1β

## Abstract

Although 3-aminopropyl functionalized magnesium phyllosilicate nanoparticles (hereafter aminoclay nanoparticles, ACNs) are well-known nanomaterials employed as drug carriers, their effects on immune cells remain unclear. To address this issue, we explored murine dendritic cells (DCs) as these cells belong to the innate arm of the immune system and function as antigen-presenting cells to elicit adaptive immune responses. We examined the in vitro effects of ACNs on DCs isolated from B6 mice. ACN treatment significantly down-regulated the expression of inflammasome-related markers, including NLRP3, caspase-1, and IL1β. The ACNs-induced anti-inflammatory DC phenotype was further confirmed by down-regulation of the AKT/mTOR/HIF1α signaling pathway. Such anti-inflammatory effects of ACNs on DCs occurred independently of DC subtypes. To document the effects of ACNs on DCs more clearly, we examined their anti-inflammatory effects on lipopolysaccharide (LPS)-activated DCs. As expected, excessive inflammatory responses (increased mitochondrial ROS and Th1-type cytokines such as IL12 and IL1β) of LPS-activated DCs were dramatically attenuated by ACN treatment. Furthermore, ACNs down-regulated IFNγ production by antigen-specific CD4^+^ T cells, which is consistent with a reduced inflammatory phenotype of DCs. Overall, our results provide support for employing ACNs as drug delivery materials with therapeutic potential to control inflammatory disorders.

## 1. Introduction

A drug delivery system (DDS) is essential for efficient drug delivery to the desired target location and is required to elicit optimal drug efficacy. Aminoclay nanoparticles (ACNs), one of the drug carriers that can be orally administered, are synthetic organic–inorganic layered materials delaminated to water-soluble cationic nanosheets in water [1]. ACNs with a positive charge can interact with negatively charged drugs to produce drug–ACN complexes, which enhance the dissolution and absorption rates of poorly water-soluble drugs such as flurbiprofen and telmisartan in the gastrointestinal tract [2,3]. Furthermore, ACN-based carrier systems can be employed for delivering insulin to the intestine [4]. Furthermore, due to their charged properties, ACNs enable the intracellular delivery of genetic materials (i.e., plasmid DNA and viral vectors) [5,6]. In addition, it has recently been reported that ACN-based carrier systems can effectively deliver Infliximab (a tumor necrosis factor [TNF]α-blocking chimeric monoclonal antibody) to the intestine and subsequently prevent dextran sulfate sodium-induced colitis in mice [7].

Most studies on ACNs have focused on their delivery effects as nanocarriers but little is known about their physiological effects in recipients. In particular, the effects of ACNs on the immune system remain unexplored. Among immune cells, dendritic cells (DCs), as innate antigen-presenting cells (APCs), play an essential role in initiating adaptive immune responses. They take up antigens and present them to CD4^+^ T cells that mediate adaptive immune responses, such as antibody production against pathogens [8]. In addition, DC-derived IL12 has been shown to play a pivotal role in the differentiation of T helper (Th)1 cells [9,10]. Upon recognizing danger signals, DCs trigger inflammatory responses which are mediated by the inflammasome (i.e., NLRP3) complex to produce IL1β [11]. Compared with macrophages, DCs possess a superior capacity to produce IL1β and NLRP3 in response to lipopolysaccharide (LPS) stimulation. These pro-inflammatory effects of DCs are independent of the ATP-P2X7 receptor axis [12]. During inflammatory immune responses, the AKT/mTOR/HIF1α signaling axis and mtROS (mitochondrial reactive oxygen species) modulate the maturation, activation, and survival of DCs [13,14]. DCs can be divided into three subpopulations, which consist of plasmacytoid DCs (pDCs), myeloid DCs (mDCs), and conventional DCs (cDCs). pDCs can produce type I interferons (IFNs) in response to Toll-like receptor (TLR)7 and TLR9 ligands. cDCs are superior in presenting exogenous antigens to naive CD8^+^ T cells, but mDCs have a high intrinsic capacity for MHC class II-mediated antigen presentation. Furthermore, based on their functional differences DCs are classified into two groups, IRF8^+^ DC1 driving Th1 responses and IRF4^+^ DC2 driving Th2 responses [15,16].

In this study, we employed murine DCs to investigate the in vitro immunological effects of ACNs. For this purpose, we examined whether ACN treatment modulates DC phenotypes (i.e., inflammasome-related markers and AKT/mTOR/HIF1α signaling pathway) and exhibits divergent effects on distinct DC subtypes. To test the modulatory effect of ACNs on LPS-activated DCs, we analyzed the production of inflammatory cytokines (i.e., IL12 and IL1β), the expression of DC1 and DC2 markers (i.e., IRF8 and IRF4, respectively), and the production of mtROS by DCs upon LPS challenge. We next examined the effect of ACNs on the capacity of fluorescently labeled LPS to bind to the cell surface of DCs. Furthermore, we investigated whether ACNs can affect antigen-specific adaptive immune responses using the ovalbumin (OVA)-specific DO11.10 T cell receptor (TCR) transgenic (Tg) mouse system and KJ1-26 mAb specific to DO11.10 TCR-expressing T cells.

## 2. Results

### 2.1. ACN Treatment Attenuates Basal Levels of Immunogenicity of Resting DCs In Vitro

**Figure 1:**
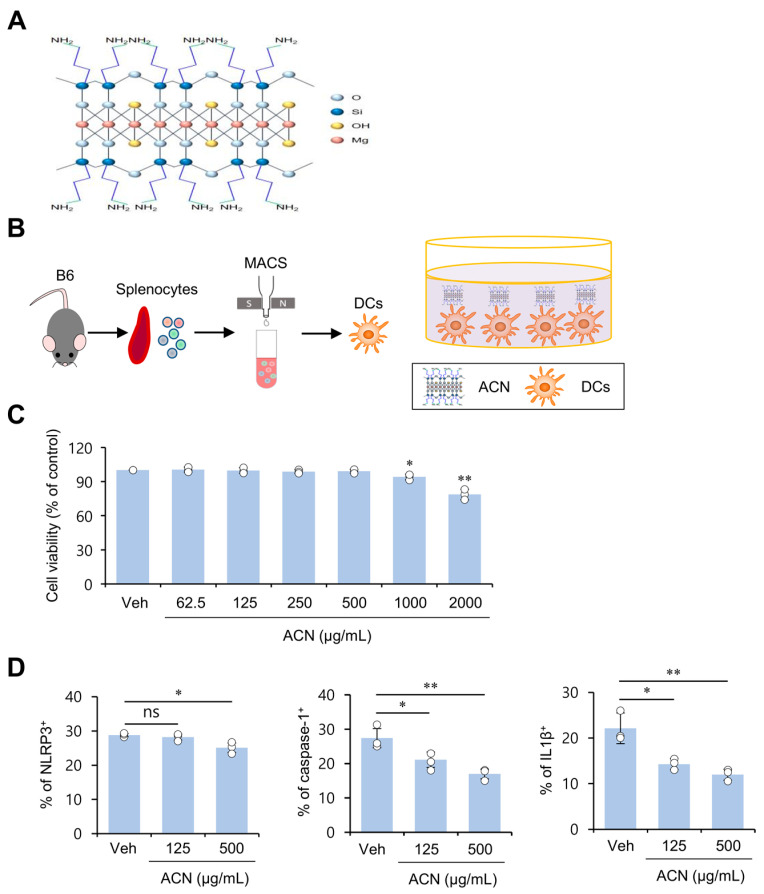
ACN treatment attenuates basal levels of the immunogenicity of resting DCs in vitro. (**A**) Schematic illustration for the molecular structure of ACNs. (**B**,**C**) Splenic CD11c^+^ DCs were purified from B6 mice using a MACS system, and DCs were cultured with ACNs (62.5, 125, 250, 500, 1000, and 2000 μg/mL) for 16 h. (**C**) The frequency of viable cells (annexin-V^−^7AAD^−^) among DCs was assessed using flow cytometric analysis. (**D**) Splenic DCs were cultured with ACNs (125 and 500 μg/mL). 16 h later, intracellular expression of NLRP3, caspase-1, and IL1β in DCs was assessed via flow cytometry. The mean values ± SD (*n* = 3; per group in the experiment; Student’s *t*-test; * *p* < 0.05, ** *p* < 0.01) are shown. One representative experiment of two experiments is shown. ns, not significant.

ACNs are layered materials composed of magnesium phyllosilicate functionalized with aminopropyl groups [1,2] (Figure 1A). Although it has been previously reported that ACNs exhibit low toxicity in human lung and skin fibroblast cell lines [1], whether ACNs can modulate the immune system remains largely unknown. To address this issue, we employed murine DCs since these cells are critical in initiating immune responses. First, we examined whether ACN treatment can influence cell viability and induce DCs to express NLRP3 inflammasome markers (i.e., NLRP3, caspase-1, and IL1β) (Figure 1B). ACNs were not cytotoxic to DCs at a concentration as high as 500 μg/mL but exhibited cytotoxicity at a concentration above 1000 μg/mL (Figure 1C). In addition, ACN stimulation decreased basal levels of intracellular NLRP3, caspase-1, and IL1β expression by DCs in a dose-dependent manner, implying that ACNs can decrease the basal levels of the immunogenicity of DCs (Figure 1D). Thus, our findings demonstrate that ACNs may be functional biomaterials with anti-inflammatory properties, not just inert nanocarriers.

### 2.2. ACN Treatment Down-Regulates Basal AKT/mTOR/HIF1α Signaling in DCs in a MyD88-Independent Manner In Vitro

**Figure 2:**
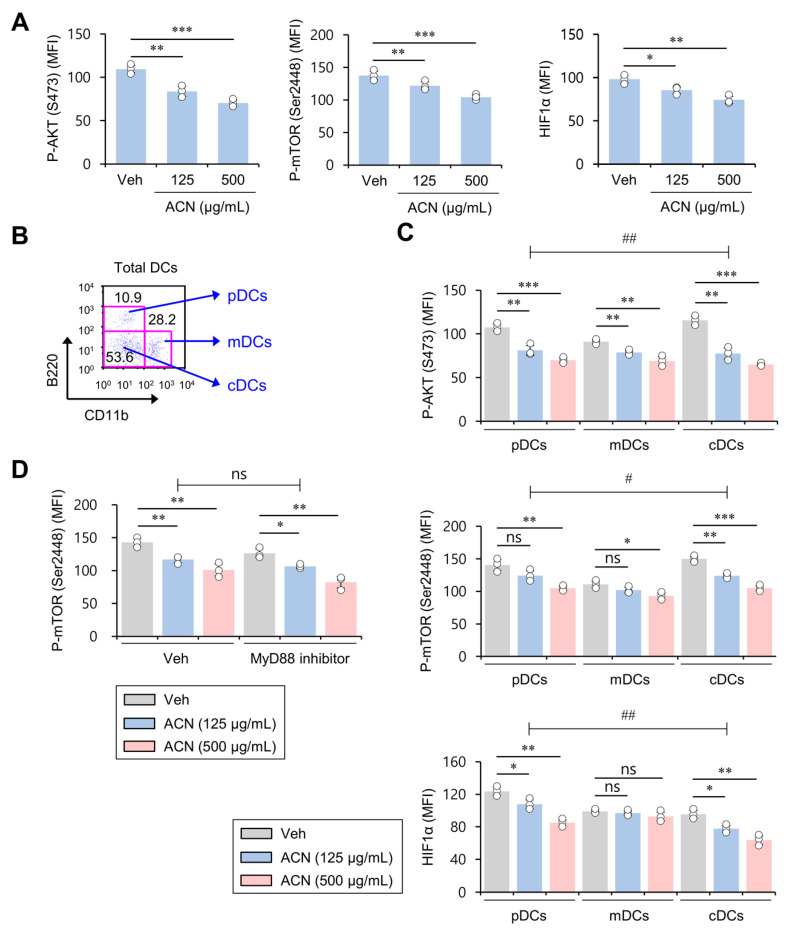
ACN treatment down-regulates basal AKT/mTOR/HIF1α signaling in DCs in a MyD88-independent fashion in vitro. (**A**–**C**) Splenic CD11c^+^ DCs were purified from B6 mice using a MACS system, and DCs were cultured with ACNs (125 and 500 μg/mL) for 16 h. (**A**) Intracellular expression of P-AKT, P-mTOR, and HIF1α in DCs was assessed via flow cytometry. (**B**) Splenic DCs were cultured with ACNs (125 and 500 μg/mL). The percentage of three subsets (pDCs (CD11b^−^B220^+^), mDCs (CD11b^+^B220^−^), and cDCs (CD11b^−^B220^−^)) among splenic DCs was evaluated using flow cytometry. (**C**) Intracellular expression of P-AKT, P-mTOR, and HIF1α was determined in DC subpopulations (pDCs, mDCs, and cDCs) via flow cytometry. Two-way ANOVA (ACN × MyD88 inhibitor) showed an interaction between these two factors (^#^ *p* < 0.05, ^##^ *p* < 0.01). (**D**) Splenic DCs were cultured for 16 h with either vehicle (Veh) or MyD88 inhibitor (T6167923; 500 μM) in the absence or presence of ACNs (125 and 500 μg/mL). Intracellular expression of P-mTOR in DCs was assessed via flow cytometry. Two-way ANOVA (subpopulation × ACN) showed an interaction between these two factors. The mean values ± SD (*n* = 3; per group in the experiment; Student’s *t*-test; * *p* < 0.05, ** *p* < 0.01, *** *p* < 0.001) are shown. One representative experiment of three experiments is shown. ns, not significant.

It has been reported that the AKT/mTOR/HIF1α signaling pathway is involved in the inflammatory processes of DCs [17,18]. Thus, to investigate whether the anti-inflammatory effects of ACNs on DCs are mediated by AKT/mTOR/HIF1α signaling, we compared their intracellular levels in ACN-treated DCs with those in vehicle (Veh)-treated controls. ACNs exhibited dose-dependent inhibitory effects on AKT/mTOR/HIF1α activity (Figure 2A). Based on their expression profile of the cell surface markers B220 and CD11b, DCs can be divided into B220^+^CD11b^−^ pDCs, B220^−^CD11b^+^ mDCs, and B220^−^CD11b^−^ cDCs [15,16] (Figure 2B). Thus, we examined the expression of P-AKT, P-mTOR, and HIF1α in these three subpopulations. Interestingly, phosphorylation of AKT and mTOR, but not HIF1α, in most DC subsets was significantly reduced by ACN treatment in a dose-dependent manner. However, mDCs were much less affected by ACN treatment than pDCs and cDCs (Figure 2C). Because it has been previously demonstrated that MyD88 signaling is also required for anti-inflammatory responses [19], we examined whether MyD88 signaling is involved in reducing P-mTOR expression in ACN-triggered DCs. However, we found that MyD88 inhibitor treatment did not significantly influence decreased P-mTOR expression by ACN treatment, implying the MyD88-independent anti-inflammatory effects of ACNs (Figure 2D). These results identify the regulation of the AKT/mTOR/HIF1α signaling pathway in the suppressive effects of ACN treatment on DC activation.

### 2.3. ACN Treatment Attenuates the Pro-Inflammatory Response of DCs in Response to In Vitro LPS Treatment

**Figure 3:**
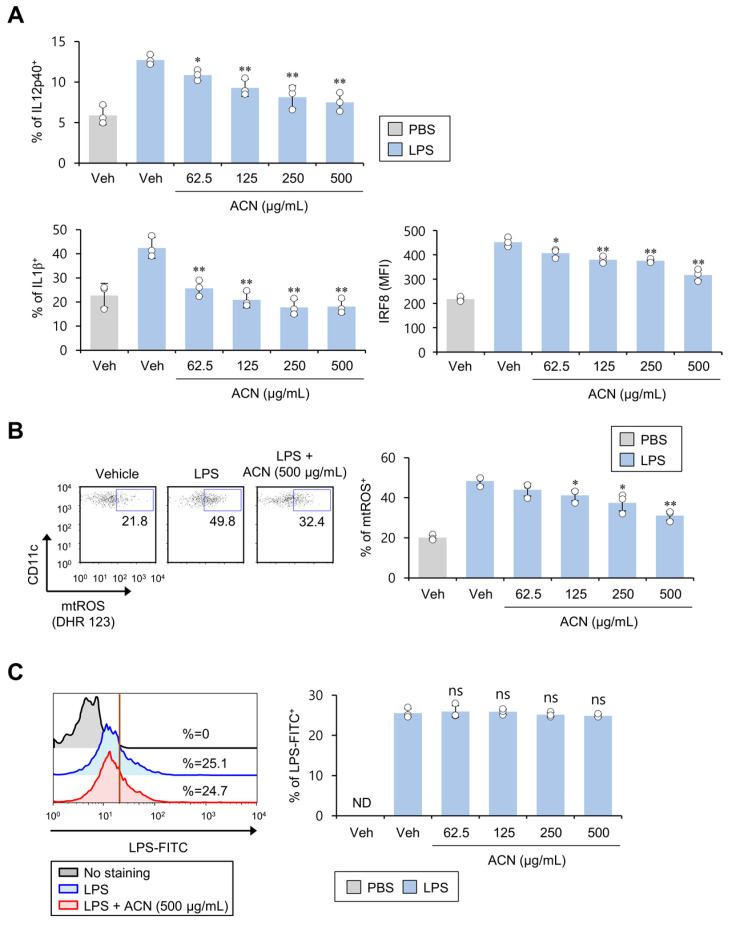
ACN treatment attenuates the pro-inflammatory response of DCs in response to in vitro LPS stimulation. (**A**,**B**) Splenic DCs were cultured for 16 h with ACNs (62.5, 125, 250, and 500 μg/mL) in the presence of LPS (1 μg/mL). (**A**) Intracellular IL12p40, IL1β, and IRF8 levels in DCs were measured using flow cytometry. (**B**) MtROS production was determined by gating on DHR 123^+^ populations in DCs. Left, representative FACS plot; right, summary figures. (**C**) Splenic CD11c^+^ DCs were isolated from B6 mice; these cells were subsequently cultured with ACNs (62.5, 125, 250, and 500 μg/mL) in the presence of LPS-FITC (1 μg/mL) and, 1 h later, the binding of LPS-FITC to DCs was measured using flow cytometry. Left, representative FACS histogram; right, summary figures. The mean values ± SD (*n* = 3; per group in the experiment; Student’s *t*-test; * *p* < 0.05, ** *p* < 0.01) are shown. One representative experiment of two experiments is shown. ns, not significant.

Upon LPS stimulation, DCs produce IL1β via the NLRP3 inflammasome [12]. An increase in IRF8 induces DCs to differentiate toward the pro-inflammatory DC1 phenotype [20]. Therefore, we investigated whether ACN treatment alters the LPS-stimulated pro-inflammatory responses of DCs. To address this issue, we analyzed inflammatory cytokines (e.g., IL12 and IL1β) and transcription factor IRF8 in DCs upon LPS challenge. As expected, the ACN-induced, dose-dependent suppression of IL12 and IRF8 production by DCs strongly inhibited IL1β production in DCs even at a low concentration (Figure 3A). Since imiquimod-induced mtROS is strongly associated with an increase in IL1β expression in DCs [14], we examined whether ACN treatment can influence mtROS levels in DCs in response to LPS stimulation. To test this possibility, mtROS production was examined using DHR 123 assay in ACN-treated DCs in the presence of LPS. We found that ACN stimulation induces a potent dose-dependent inhibitory effect on mtROS production by LPS-activated DCs (Figure 3B). Since a previous study proposed the blockade of LPS binding to the cell membrane as a therapeutic approach to attenuate pro-inflammatory signaling pathways [21,22], we explored whether ACNs affect the binding of LPS to DCs. To address this issue, fluorescently labeled LPS (LPS-FITC conjugate) was incubated with ACNs for one hour. However, inhibition of LPS binding to the cell surface was not associated with ACN-induced suppression of LPS-mediated DC activation (Figure 3C). These results indicate that the ACN-treatment-mediated decrease in IL1β secretion in LPS-stimulated DCs might be associated with down-regulated mtROS production.

### 2.4. ACN Treatment Inhibits Antigen-Specific Th1 Polarization Independently of TCR Signaling

**Figure 4:**
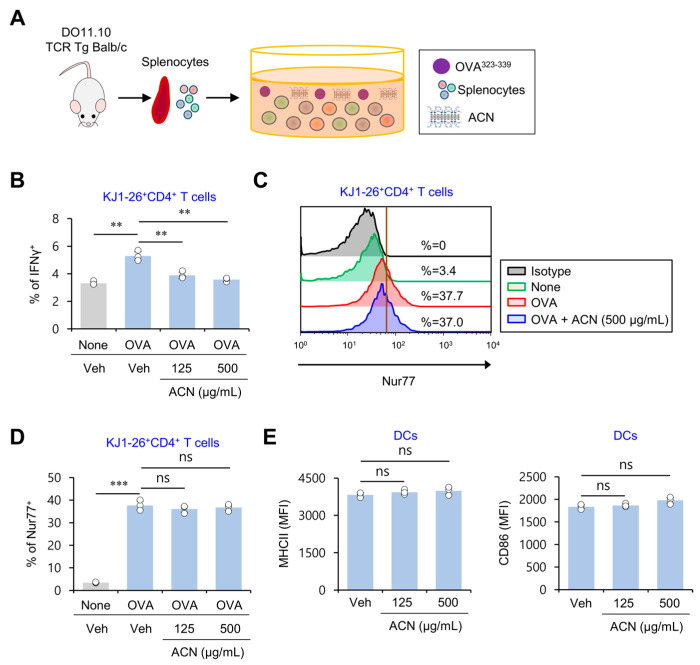
ACN treatment inhibits antigen-specific Th1 polarization independent of TCR signaling strength. (**A**–**D**) Splenocytes isolated from DO11.10 TCR Tg Balb/c mice were cultured with either Veh or ACNs (125 and 500 μg/mL) in the presence of OVA peptide_323–339_ (10 μg/mL) for three days. (**B**) Intracellular IFNγ production in CD4^+^ T cells (KJ1-26^+^CD3ε^+^CD4^+^) was assessed via flow cytometry. (**C**,**D**) Intracellular Nur77 expression in CD4^+^ T cells (KJ1-26^+^CD3ε^+^CD4^+^) was assessed via flow cytometry. (**C**), representative FACS plot; (**D**), summary figures. (**E**) Splenic CD11c^+^ DCs were purified from B6 mice using a MACS system and subsequently cultured with ACNs (125 and 500 μg/mL). 16 h later, surface MHCII and CD86 expression in DCs was measured using flow cytometry. The mean values ± SD (*n* = 3; per group in the experiment; Student’s *t*-test; ** *p* < 0.01, *** *p* < 0.001) are shown. One representative experiment of two experiments is shown. ns, not significant.

The cytokine IL12 produced by DCs has a central role in the induction of Th1 cell differentiation [9]. Since our findings showed that ACN treatment suppresses IL12 production elicited by LPS-activated splenic DCs, we evaluated whether ACN treatment influences the Th1 polarization of antigen-specific T cells. For this purpose, DO11.10 OVA-specific TCR Tg mice were employed. First, splenocytes from these mice were isolated and subsequently activated by adding OVA_323–339_ peptide in the presence of either Veh or ACNs in vitro. Then, we examined the expression of a signature Th1 cytokine (i.e., IFNγ) and a marker of TCR signaling strength (i.e., Nur77) in KJ1-26^+^CD4^+^ T cells using flow cytometry (Figure 4A). As expected, splenic CD4^+^ T cells treated with OVA plus ACNs produced significantly lower levels of IFNγ. However, considering that these cells expressed comparable levels of Nur77 compared with OVA-treated controls, the inhibitory effect of ACN treatment on Th1 differentiation appears not to be caused by alterations in TCR signaling strength (Figure 4B–D). Consistent with this observation, ACN treatment did not influence the expression levels of the antigen-presenting molecule MHC II and the costimulatory molecule CD86, which are both required for T cell activation (Figure 4E). These results identify ACNs as biomaterials that can modulate Th1 adaptive immunity.

## 3. Discussion

Although previous studies have reported ACNs as desirable delivery nanomaterials for hydrophobic drugs [2,3], their effects on innate immune cells such as DCs have remained unexplored. Herein, we investigated whether ACN treatment can influence inflammatory responses of DCs and identified their inhibitory effects on inflammatory immune responses mediated by both resting and LPS-stimulated DCs.

DCs and macrophages are professional APCs. However, DCs show a more superior APC function than macrophages, whereas macrophages exhibit more potent phagocytic activity against microorganisms than DCs [23]. In addition, DCs and macrophages display distinct features in their tissue residence, heterogeneity, and metabolism [24]. While M1 macrophages promote inflammatory responses via the production of “pro-inflammatory” cytokines (i.e., TNFα, IL12, and IL6), M2 macrophages enhance wound repair and tissue regeneration via the production of “anti-inflammatory” cytokines (i.e., IL10) [25]. Since ACN treatment significantly inhibited excessive production of IL12 by LPS-stimulated macrophages in a dose-dependent manner (Figure S1, Supplementary Materials), it will be exciting to investigate further whether ACNs modulate M1 and M2 macrophage polarization.

Polyamines (e.g., putrescine, spermidine, and spermine) are organic cationic compounds with more than two amino groups. It has been previously reported that polyamines possess immunomodulatory properties. For example, putrescine treatment suppressed the expression of TNFα, IL6, and IL8 in intestinal porcine epithelial cell lines after LPS stimulation in vitro [26]. In addition, spermidine and spermine inhibit LPS-stimulated pro-inflammatory cytokine release in murine microglial cell lines and human mononuclear cells, respectively [27,28]. In particular, spermidine suppresses excessive production of TNFα, IL6, and IL12p40 by DCs following TLR7 ligand stimulation [29]. Furthermore, since positively charged polyamines interact with negatively charged molecules, including nucleic acids, acidic proteins, phospholipids, and ATP [30], these polyamines can act as free radical scavengers neutralizing ROS, negatively charged molecules involved in inflammatory responses [31]. Positively charged liposomes show higher uptake by macrophages than neutral and negatively charged liposomes [32]. Moreover, it has been reported that cationic liposomes, but not anionic liposomes, inhibit the production of nitric oxide (NO) and TNFα by LPS-stimulated macrophages and suppress carrageenan-induced footpad inflammation [33]. These previous reports indicate that the positively charged ACNs show more significant interaction with professional APCs, such as macrophages and DCs, consequently inhibiting the inflammatory response of these cells. Based on the previous reports and current findings, it will be interesting to examine whether amine functional groups of ACNs might play important roles in clearing negatively charged endogenous inflammatory molecules via charge-to-charge interactions.

Although IL4-producing immune cells (e.g., basophils, mast cells, and Th2 cells) play a crucial role in Th2-mediated immune diseases such as atopic dermatitis (AD) [10,34,35,36], DCs are also essential because they can initiate immune responses and modulate CD4^+^ T cell polarization [37]. Furthermore, IRF4-expressing DCs promote Th2 differentiation, ultimately resulting in the development of Th2-mediated allergic responses [38]. In addition, we found that ACNs can significantly attenuate the increased IRF4 expression in LPS-stimulated DCs (Figure S2, Supplementary Materials). These findings suggest that ACN-mediated down-regulation of IRF4 expression in DCs might contribute to inhibiting the progression of allergic diseases such as AD.

In conclusion, our results show that ACN treatment directly attenuates inflammatory responses in LPS-induced DCs. However, what molecules (or receptors) are responsible for interacting with ACNs remains unclear. Thus, it will be interesting to investigate the receptors expressed on the DCs involved in these anti-inflammatory immune responses mediated by ACNs via binding with negatively charged bioactive molecules such as ROS.

## 4. Materials and Methods

### 4.1. Study Design

This study was designed to determine the effect of ACNs on LPS-induced DC activation. To address this issue, magnetically activated cell sorting (MACS)-purified DCs were treated with ACNs and LPS for 16 h and, subsequently, DCs were harvested and further analyzed using flow cytometry.

### 4.2. Mice and Reagents

WT B6 mice were purchased from Jung Ang Lab Animal Inc. (Seoul, Republic of Korea). IL12p40 reporter (Yet40) B6 mice were provided by Dr. R. Locksley (University of California at San Francisco, CA, USA). DO11.10 OVA-specific TCR Tg mice used in this study are of the Balb/c genetic background. These mice were maintained at Sejong University and were used for experiments at 6–12 weeks of age. They were maintained on a 12 h light/12 h dark cycle in a temperature-controlled barrier facility with free access to food and water. Mice were fed a γ-irradiated sterile diet and provided with autoclaved tap water. Age- and sex-matched mice were used for all experiments. The animal experiments were approved by the Institutional Animal Care and Use Committee of Sejong University (SJ-20190301). Unlabeled LPS and FITC-labeled LPS derived from *Escherichia coli* (serotype 0111:B4) were purchased from Sigma-Aldrich (St. Louis, MO, USA). MyD88 inhibitor (T6167923) was purchased from Toronto Research Chemicals (Martin Ross Ave, Toronto, Canada). OVA peptide_323–339_ (ISQAVHAAHAEINEAGR) was synthesized by Peptron Inc. (Daejeon, Republic of Korea).

### 4.3. Preparation of ACNs

ACNs were synthesized as described in a previous report [2]. In brief, 3-aminopropyl triethoxysilane (1.3 mL) was added dropwise with rapid stirring to magnesium chloride (0.84 g) dissolved in ethanol (20 mL). A white precipitate formed almost immediately and was stirred overnight. The resulting product was separated via centrifugation, washed with ethanol (3 × 50 mL), and dried under vacuum at 40 °C. For the exfoliation of the obtained ACNs, the bulk powder was dispersed in water and subjected to ultrasonication for 10 min.

### 4.4. Cell Isolation and Culture

Splenic CD11c^+^ DCs were isolated from B6 mice using a MACS system (Miltenyi Biotec, Bergisch Gladbach, Germany), following the manufacturer’s instructions [39]. CD11c^+^ DCs were enriched >94% after MACS. Primary cells were cultured in RPMI 1640 (Gibco BRL, Gaithersburg, MD, USA) culture media supplemented with 10% FBS, 10 mM HEPES, 2 mM l-glutamine, 100 units/mL penicillin-streptomycin, and 5 μM 2-mercaptoethanol.

### 4.5. Isolation of Peritoneal Macrophages

Yet40 B6 mice were intraperitoneally (i.p.) injected with 2 mL of 4% thioglycolate broth (Sigma, St. Louis, MO, USA) in deionized water. Five days later, peritoneal macrophages were harvested from peritoneal lavage fluid and were subsequently plated on 24-well plates to select adherent macrophages. Two hours later, the nonadherent cells were removed via washing with warm RPMI medium.

### 4.6. Flow Cytometry

The following monoclonal antibodies (mAbs) were obtained from BD Biosciences (San Jose, CA, USA): fluorescein isothiocyanate (FITC)-, phycoerythrin (PE)-Cy7-, or allophycocyanin (APC)-conjugated anti-CD11c (clone HL3); FITC-, PE-Cy7-, or APC-conjugated anti-CD3ε (clone 145-2C11); FITC-, PE-Cy7-, or APC-conjugated anti-CD4 (clone RM4-5); FITC- or PE-Cy7-conjugated anti-CD11b (clone M1/70); FITC- or PE-Cy7-conjugated anti-B220 (clone RA3-6B2); PE-conjugated anti-IL12p40 (clone C15.6); and PE-conjugated anti-CD86 (clone GL1). The following mAbs from Thermo Fisher Scientific (Waltham, MA, USA) were used: FITC-, PE-Cy7-, or APC-conjugated anti-MHCII (clone M5/114.15.2); FITC- or PE-Cy7-conjugated anti-DO11.10 TCR (clone KJ1-26); PE-conjugated anti-IL1β (clone NJTEN3); PE-conjugated anti-Phospho-mTOR (Ser2448) (clone MRRBY); PE-conjugated anti-IRF4 (clone 3E4); PE-conjugated anti-IRF8 (clone V3GYWCH); PE-conjugated anti-Nur77 (clone 12.14); and PE-conjugated anti-IFNγ (clone XMG1.2). The following mAb from LifeSpan BioSciences (Seattle, WA, USA) was used: PE-conjugated anti-caspase-1 (polyclonal aa119-296). The following mAbs from R&D systems (Minneapolis, MN, USA) were used: PE-conjugated anti-NLRP3 (clone 768319); PE-conjugated anti-Phospho-AKT (Ser473) (clone 545007); and PE-conjugated anti-HIF1α (clone 241812). Cells were harvested and washed twice with cold 0.5% BSA-containing PBS (FACS buffer) for staining surface markers. For blocking Fc receptors, the cells were incubated with anti-CD16/CD32 mAbs (clone 2.4G2) on ice for 10 min and subsequently stained with fluorescently labeled mAbs. Flow cytometric data were acquired using a FACSCalibur flow cytometer (Becton Dickson, San Jose, CA, USA) and analyzed using FlowJo software (Tree Star Inc., Ashland, OR, USA).

### 4.7. Intracellular Cytokine Staining

For intracellular staining, splenocytes were incubated with brefeldin A, an intracellular protein transport inhibitor (10 μg/mL), in RPMI medium for 2 h at 37 °C. The cells were stained for cell surface markers, fixed with 1% paraformaldehyde, washed once with cold FACS buffer, and permeabilized with 0.5% saponin. The permeabilized cells were then stained for an additional 30 min at room temperature with the indicated mAbs (PE-conjugated anti-NLRP3, anti-caspase-1, anti-IL1β, anti-HIF1α, anti-IL12p40, anti-IRF8, anti-IRF4, anti-Nur77, anti-IFNγ, or PE-conjugated isotype control rat IgG mAbs) [40]. More than 5000 cells per sample were acquired using a FACSCalibur, and the data were analyzed using the FlowJo software package (Tree Star Inc., Ashland, OR, USA).

### 4.8. Determination of Mitochondrial ROS

Splenic DCs were cultured with ACNs in the presence of LPS (1 μg/mL) for 16 h, followed by incubation with 1 μM Dihydrorhodamine 123 (DHR 123) (Sigma, St. Louis, MO, USA) at 37 °C for 30 min. ROS fluorescence intensity was determined using flow cytometry.

### 4.9. Phosphoflow Analysis of Protein Phosphorylation Levels

Cells were fixed in pre-warmed Fix Buffer I (BD Phosflow™ Cat. No. 557870) for 10 min at 37 °C. Immediately after washing with cold PBS, permeabilization was performed with cold PhosflowPermBuffer II (BD Phosflow™ Cat. No. 558050) for 30 min on ice. Next, the cells were washed twice with staining buffer (1× PBS with 2% FBS) for 10 min and subsequently stained with PE-conjugated anti-Phospho-mTOR (Ser2448) and anti-Phospho-AKT (Ser473) mAb in staining buffer for 30 min at room temperature (RT). More than 5000 cells per sample were acquired using the FACSCalibur and analyzed with the FlowJo software package.

### 4.10. Statistical Analysis

Statistical significance was determined using Excel (Microsoft, Redmond, WA, USA). Student’s *t*-test was performed to compare two groups (* *p* < 0.05, ** *p* < 0.01, and *** *p* < 0.001 were considered significant in the Student’s *t*-test). Two-way ANOVA analysis was carried out using the VassarStats (http://vassarstats.net/anova2u.html) (accessed on 22 February 2022) (# *p* < 0.05, ## *p* < 0.01, and ### *p* < 0.001 were considered significant in the two-way ANOVA).

## Data Availability

The data are available from the corresponding author on reasonable request.

## Supplementary Materials

The following supporting information can be downloaded at www.mdpi.com/article/10.3390/molecules27248743/s1, Figure S1: ACN treatment attenuates IL12 production by macrophages in response to in vitro LPS stimulation; Figure S2: ACN treatment attenuates IRF4 expression in DCs upon in vitro LPS stimulation.
