# Supplementary material for: Aminoclay Nanoparticles Induce Anti-Inflammatory Dendritic Cells to Attenuate LPS-Elicited Pro-Inflammatory Immune Responses
Source: Molecules. 2022 Dec 9;27(24):8743. doi: 10.3390/molecules27248743 (PMC9787634; doi:10.3390/molecules27248743)
Supplement: Supplementary file 1 [file molecules-27-08743-s001.zip › molecules-2030244-supplementary.pdf]

Supplementary Materials

Aminoclay Nanoparticles Induce Anti-Inflammatory Dendritic Cells to Attenuate LPS-Elicited Pro-Inflammatory Immune Responses

This document includes Supplementary Figures S1–S2.

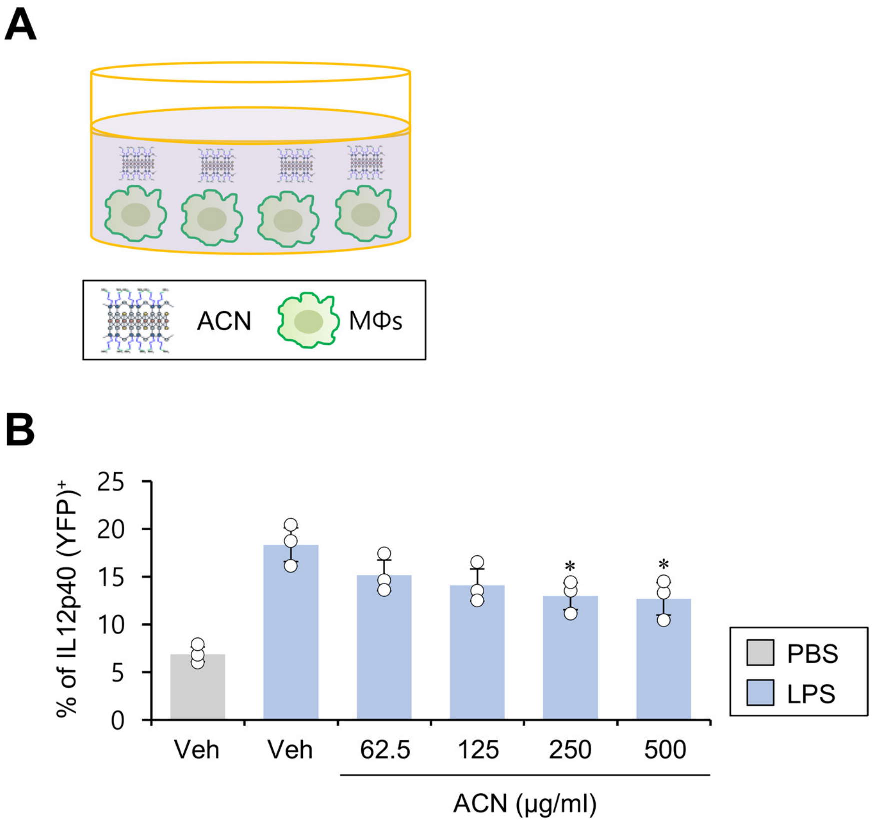

Figure S1. ACN treatment attenuates IL12 production by macrophages in response to in vitro LPS stimulation.

(A-B) Peritoneal macrophages isolated from Yet40 B6 mice were primed for 2 hrs with IFNγ (20 ng/mL). IFNγ-primed macrophages were cultured for 16 hrs with ACN (62.5, 125, 250, and 500 μg/mL) in the presence of LPS (1 μg/mL). (B) IL12p40 (YFP) production in splenic DCs ($CD11c^{+}$) from each experimental group was analyzed by flow cytometry. The mean values ± SD are shown (n = 3 per group in the experiment; Student's t-test; *$p < 0.05$). One representative experiment of two experiments is shown.

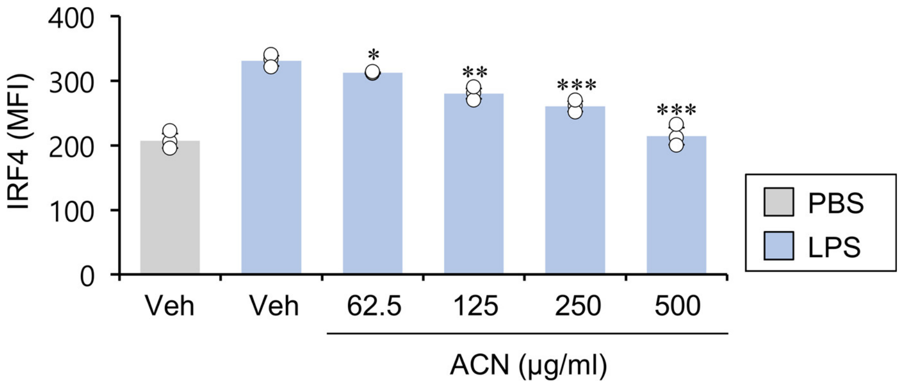

Figure S2. ACN treatment attenuates IRF4 expression in DCs upon *in vitro* LPS stimulation.

Splenic DCs were cultured for 16 hrs with ACN (62.5, 125, 250, and 500 μg/mL) in the presence of LPS (1 μg/mL). Intracellular IRF4 levels in DCs were measured by flow cytometry. The mean values ± SD (n = 3; per group in the experiment; Student's t-test; *$p < 0.05$, **$p < 0.01$, ***$p < 0.001$) are shown. One representative experiment of two experiments is shown.
